# Analysis of Differences in Volatile Components of Rucheng Baimao (*Camellia pubescens*) Black Tea in Different Seasons

**DOI:** 10.3390/foods14050763

**Published:** 2025-02-24

**Authors:** Junye Zhu, Yuebin Zhou, Haitao Wen

**Affiliations:** 1 Key Laboratory of Tea Science of Ministry of Education, Hunan Agricultural University, Changsha 410128, China; zjy1287546014@163.com (J.Z.); chowyuebin@163.com (Y.Z.); 2 National Research Center of Engineering and Technology for Utilization of Botanical Functional Ingredients, Hunan Agricultural University, Changsha 410128, China; 3 Co-Innovation Center of Education Ministry for Utilization of Botanical Functional Ingredients, Hunan Agricultural University, Changsha 410128, China; 4 Key Laboratory for Evaluation and Utilization of Gene Resources of Horticultural Crops, Ministry of Agriculture and Rural Affairs of China, Hunan Agricultural University, Changsha 410128, China; 5 Huangpu Innovation Research Institute, Hunan Agricultural University, Guangzhou 510700, China

**Keywords:** Rucheng Baimao, black tea, volatile components, season

## Abstract

At present, there are few studies on seasonal differences in the aroma quality and volatile components of Rucheng Baimao (*Camellia pubescens*) black tea. In this study, sensory evaluation and volatile component analysis were carried out on one sample of Rucheng Baimao black tea corresponding to spring, summer, and autumn, respectively. The results of sensory evaluation showed that the black teas of all three seasons had floral aromas. However, the aroma quality of spring black tea was the best, followed by that of autumn black tea, and summer black tea was the worst. The analysis of volatile components showed that alcohols, esters, and alkanes were the main substance categories. In addition, the results of the aroma index were consistent with those of the sensory evaluation, indicating that spring black tea had the best aroma quality, followed by autumn black tea and then summer black tea. Eleven key differential volatile components were screened by combining PLS-DA analysis (VIP > 1, *p* < 0.05) and rOAV > 1. Among them, geraniol, methyl salicylate, nonanal, and (*E*)-citral accumulated the most in spring black tea, linalool, phenylacetaldehyde, benzaldehyde, phenethyl alcohol, benzyl alcohol, and *β*-ionone accumulated the most in summer black tea, and *trans*-nerolidol accumulated the most in autumn black tea. This study aims to provide a theoretical reference for the regulation of the aroma quality of Rucheng Baimao black tea.

## 1. Introduction

Tea is one of the three major non-alcoholic beverages in the world and is widely popular due to its potential health benefits and unique flavor profiles [1,2]. Black tea, a fully fermented tea, is one of the six major tea categories in China. It is characterized by red-colored liquor, red- hued leaves, a sweet taste, and a mellow flavor. Black tea is the most favored tea type among consumers, accounting for approximately 78% of total global tea consumption [3]. Hunan is one of the primary black tea producing provinces in China, and the black tea produced there is well known for its distinct floral aroma [4,5]. Rucheng Baimao (*Camellia pubescens*), native to Rucheng County, Hunan Province, is one of the four rare wild tea tree varieties in Hunan. It is named for the white hairs that cover the surface of its leaves. It is rich in biochemical components, and is especially high in tea polyphenols and catechins, making it an excellent raw material for producing high-quality black tea [6,7,8].

Aroma is a crucial factor in evaluating the quality of black tea and significantly influences consumers’ purchasing decisions. Thus, identifying the aroma compounds in black tea has drawn the interest of many researchers. Studies have indicated that floral, sweet, and fruity aromas are typical aroma characteristics of black tea [9]. Currently, more than 600 volatile components have been detected in black tea [7]. Components such as linalool, phenethyl alcohol, phenylacetaldehyde, methyl salicylate, and geraniol have been identified as key aroma substances in black tea, they possess characteristics such as floral, fruity, and sweet aromas, and have a positive impact on the aroma quality of black tea [10,11,12]. However, the aroma quality of tea is significantly influenced by seasonal factors [13]. Due to differences in the external growth environment, including light, temperature, and humidity, the freshly picked tea leaves exhibit different enzymatic and chemical properties, which results in variations in tea quality [14].

As a high-quality and characteristic tea tree resource for making black tea in Hunan Province, Rucheng Baimao has also been explored for its volatile components. Huang et al. compared the aroma characteristics of black teas made from three characteristic tea tree varieties in Hunan. The results showed that the black tea made from Rucheng Baimao had a long-lasting floral aroma, and its volatile components were mainly alcohols with sweet and floral–fruity aromas [15]. Additionally, studies have compared the key aroma substances of ‘Baimaocha’ black tea from different origins, and the results show that methyl salicylate is the main contributor to the fresh aroma characteristic of Rucheng Baimao black tea [7]. Therefore, previous studies on the aroma quality of Rucheng Baimao black tea mostly focused on comparisons with other varieties. However, to date, no research has been reported on comparing the volatile components of Rucheng Baimao black tea in different seasons. In recent years, Rucheng County has been committed to fully exploiting the excellent characteristics of Rucheng Baimao, focusing on developing mid- to high-end black tea and white tea in late spring, summer, and autumn, with the aim of vigorously promoting the sustainable development of the tea industry in Rucheng County [16]. Therefore, exploring the differences in the volatile components of Rucheng Baimao black tea in different seasons is of great significance for regulating the aroma quality of Rucheng Baimao black tea and for the sustainable development of the tea industry. Furthermore, as the seasons for black tea production in Rucheng County are spring, summer, and autumn, this study utilizes sensory evaluation, HS-SPME-GC-MS, and combines with rOAV analysis to explore the differences in the aroma quality and volatile components of Rucheng Baimao black tea in these three seasons. The purpose is to provide a theoretical reference for regulating the aroma quality of Rucheng Baimao black tea.

## 2. Materials and Methods

### 2.1. Samples and Chemicals

Fresh samples of one bud and two tea leaves were picked in Rucheng County, Hunan Province in September 2023 (autumn), April 2024 (spring), and July 2024 (summer). Rucheng Baimao is planted in the primitive secondary forest area around Jiulongjiang of the Luoxiao Mountains in Rucheng County, Hunan Province. It is located at 25°19′–25°52′ north latitude and 113°16′–113°59′ east longitude. This area belongs to the monsoon humid climate zone transitioning from the southern subtropical zone to the central subtropical zone. The climate here is warm and humid, with an average annual temperature of 16.6 °C–17.6 °C. The processing procedures of black tea are as follows: fresh leaves → withering (withered in an indoor withering trough, the thickness of leaf spreading is 3–5 cm, and stop when the water content reaches about 58–60%) → rolling (rolling without pressure for 50 min, then with light pressure for 10 min) → fermentation (fermented in a fermentation room, with a room temperature of 28–30 °C and humidity above 85%, lasting for 6 h) → drying (primary drying: pass through a chain type dryer at 130 °C twice; cool down; final drying: use a flavor enhancing machine at 80 °C for about 2.5 h until the water content is below 5%). The spring black tea is numbered as SPBT, the summer black tea as SUBT, and the autumn black tea as AUBT. The samples were sealed in aluminum foil bags and stored in a −80 °C refrigerator for testing.

Ethyl caprate standard product (99%) was purchased from Aladdin Biochemical Technology Co., Ltd. (Shanghai, China).

### 2.2. Equipment and Apparatus

HS-SPME 20 mL vials (Anpel Experimental Technology Co., Ltd., Shanghai, China); 50/30 μm DVB/CAR/PDMS extraction fiber and headspace solid phase microextraction holder (Sigma-Aldrich Co., St. Louis, MO, USA); GC/MS-QP2010 gas chromatography mass spectrometry instrument (Kyoto Shimadzu Co., Ltd., Kyoto, Japan); n-alkanes C8-C25 (Sigma-Aldrich Co., St. Louis, MO, USA).

### 2.3. Sensory Evaluation

To analyze the aroma sensory characteristics of black tea samples, we adopted the traditional sensory evaluation method. The traditional sensory evaluation was carried out by five tea-science professionals (three men and two women, aged 25–60 years old). They all received complete tea evaluation training and had 5–40 years of tea evaluation experience, enabling them to accurately evaluate the sensory characteristics of tea. The method was carried out in accordance with the Chinese national standard GB/T 23776-2018 [17]. Briefly, 3 g of tea samples were weighed and placed in a cylindrical cup. Then, 150 mL of boiling water was added. After 5 min, the tea liquor was poured out for aroma evaluation and scoring. The sensory scoring standard also referred to the method of the Chinese national standard [17], using a 100-point scale. Specifically, it was divided into three grades. The first grade was 90–99 points, the second grade was 80–89 points, and the third grade was 70–79 points. The results were the average scores of the five evaluators.

### 2.4. Detection of Volatile Components

The detection method for volatile components referred to our previously published research [18]. The volatile components of the sample were extracted using the HS-SPME method. Accurately weigh 2 g of ground tea sample and place it in a 20 mL HS-SPME vials. Add 10 μL of ethyl caprate internal standard and equilibrate in an 80 °C water bath for 10 min. Push out the extraction head and place it above the tea sample. After 50 min of absorption, immediately remove it from the GC-MS injection port and desorb for 5 min. The extraction head should be aged at 250 °C for 40 min before use. Repeat the experiment three times.

The GC conditions are as follows: chromatography column: DB-5MS (0.25 μm × 60.0 m, 0.25 mm), column temperature starting at 60 °C and maintained for 5 min, first raised to 140 °C at 3 °C/min and maintained for 5 min, then raised to 230 °C at 10 °C/min and maintained for 5 min, finally raised to 260 °C at 10 °C/min and maintained for 5 min, using 99.999% high-purity helium gas as the carrier gas, flow rate of 1.96 mL/min, no split injection, and injection port temperature of 250 °C.

The MS conditions are electron bombardment of the ion source, with an ion source temperature of 230 °C, an interface temperature of 250 °C, and a mass-to-nucleus ratio scanning range of 45~500 *m*/*z*.

### 2.5. Calculation of Relative Odor Activity Value (rOAV)

The calculation of rOAV refers to previously published research [19]. Briefly, rOAV = (relative content of volatile component)/(threshold of volatile component in water).

### 2.6. Data Processing

The identification of volatile components was based on previous research [20]. According to the total ion chromatogram from GC-MS, a search was conducted in the NIST17 mass spectral library, selecting volatile substances with a similarity of 80% or higher for qualitative analysis. The retention index (RI) of each volatile component was calculated using the retention index formula [21], combined with the retention time, and identification was further confirmed using the literature and relevant websites (https://webbook.nist.gov/chemistry/ (accessed on 15 January 2025)) for the retention index [22,23,24,25,26,27,28,29,30,31,32,33,34,35,36].

Using ethyl caprate as an internal standard, the volatile components were subjected to relative quantification. The calculation formula referenced previous research and is given by the formula: (volatile peak area × internal standard content)/(internal standard peak area × sample volume), with units expressed in μg/kg [23].

The aroma descriptions of volatile components were sourced from the VCF (https://www.vcf-online.nl/VcfHome.cfm (accessed on 15 January 2025)) and Flavornet (https://www.flavornet.org/index.html (accessed on 15 January 2025)) websites, as well as relevant studies from the literature [13,23,36,37,38,39,40,41,42,43,44,45,46,47,48,49,50,51,52].

The data are expressed as mean ± standard deviation (data from three experimental replicates). One-way ANOVA was conducted using SPSS 20 software (version 20.0, SPSS Inc., Chicago, IL, USA). Multivariate statistical analysis was performed using Simca 14.1 software (Umea, Sweden). Specifically, for PCA and HCA analyses, the UV mode was used to standardize the data, while for the PLS-DA analysis, the Par mode was employed for data standardization. The column charts were created using GraphPad Prism 9.0 software (GraphPad Software, LLC, San Diego, CA, USA). The heatmap was drawn using TBtools software (version 2.43, https://github.com/CJ-Chen/TBtools (accessed on 1 February 2023)).

## 3. Results and Discussion

### 3.1. Sensory Aroma Evaluation of Black Tea in Different Seasons

The sensory aroma evaluation of Rucheng Baimao black tea in different seasons is shown in Table 1. One-way ANOVA analysis of the sensory scores shows that the sensory scores of spring black tea are significantly higher than those of summer and autumn black tea, and the sensory scores of autumn black tea are significantly higher than those of summer black tea. The black teas of spring, summer, and autumn all possess floral aroma characteristics. However, in addition to floral–fruity and sweet aromas, the spring black tea also has a refreshing sensation, with a fresh and lively aroma that gives a pleasant feeling after being smelled. In addition, the aroma of the autumn black tea is pure and harmonious, indicating that its floral fragrance is clean and free from off-odors, and its sensory aroma quality is better than that of the summer black tea. Therefore, based on the results of sensory evaluation, the sensory aroma quality of Rucheng Baimao black tea is the best in spring, followed by autumn, and then summer.

### 3.2. Analysis of Volatile Components in Black Tea from Different Seasons

**Figure 1:**
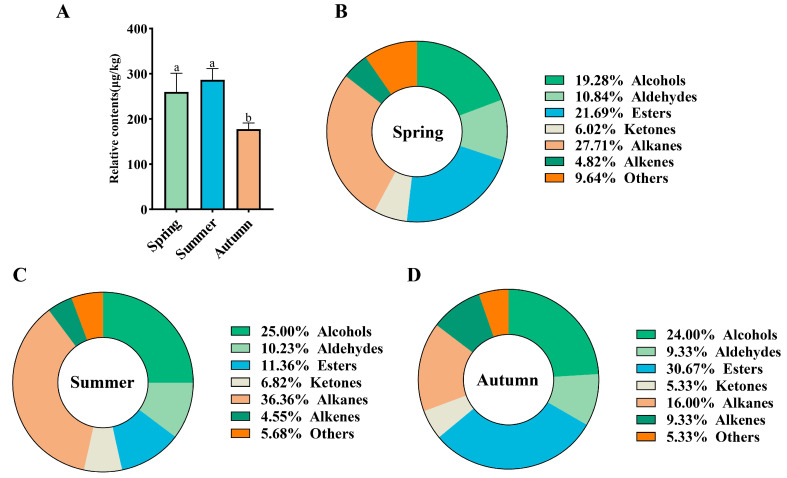
Statistical analysis of volatile components in Rucheng Baimao black tea across different seasons. (**A**) Comparison of the relative contents of volatile components in black tea in different seasons; (**B**−**D**) the proportion of volatile components in black tea in different seasons. Different lowercase letters represent significant differences (*p* < 0.05).

The volatile components of Rucheng Baimao black tea in different seasons were detected by HS-SPME-GC-MS. The results are presented in Table S1. A total of 140 volatile components were identified. Among them, 83 were detected in spring black tea, 88 in summer black tea, and 75 in autumn black tea. By comparing the relative contents of volatile components in black tea from spring, summer, and autumn, it was found that the relative content of produced black tea was the highest (286.45 μg/kg), followed by that of spring black tea (259.68 μg/kg), and the relative content of autumn black tea was the lowest (177.60 μg/kg). The relative content of black tea was significantly lower than that of spring and summer black tea (Figure 1A). The 140 volatile components can be classified into seven categories according to functional groups: alcohols (27 types), aldehydes (14 types), esters (28 types), ketones (6 types), alkanes (41 types), alkenes (13 types), and others (11 types). By comparing the proportion of different categories of volatile components in Rucheng Baimao black tea in different seasons (Figure 1B–D), it was found that alcohols, esters, and alkanes had the highest proportion in each respective season. Alkanes have no distinct odor, and alcohols and esters may be the main contributors to the aroma quality of Rucheng Baimao black tea in different seasons. The formation of alcohols is mainly related to the hydrolysis of glycosidic aroma precursor substances and the biosynthetic pathways of volatile terpenoids. Most of these substances have floral and fruity scents and have a significant impact on the aroma quality of tea [53,54]. Esters can be produced through the degradation of amino acids and fatty acids and play a crucial role in the formation of tea aroma. In particular, esters with C6–C9 carbon chains can exhibit pleasant floral, fruity, and fresh scents [10]. Ouyang et al. proposed that alcohols, aldehydes, and esters are the main substances contributing to the formation of the aroma of ‘Baimaocha black tea’, which is consistent with the results of this study [7].

**Figure 2:**
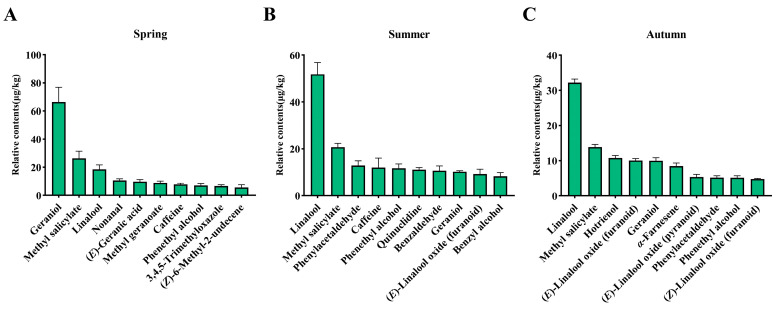
Statistics of the top 10 volatile components in Rucheng Baimao black tea in different seasons. (**A**) The top 10 volatile components in terms of content in black tea in spring; (**B**) the top 10 volatile components in terms of content in black tea in summer; (**C**) the top 10 volatile components in terms of content in black tea in autumn.

In addition, this study also statistically analyzed the top 10 volatile components in terms of relative content in black tea from different seasons (Figure 2). Geraniol had the highest relative content in spring black tea (66.34 μg/kg), which was significantly higher than that in summer (10.25 μg/kg) and autumn (9.99 μg/kg). The volatile component with the highest relative content in both summer and autumn black tea was linalool, with values of 51.83 μg/kg and 32.22 μg/kg, respectively. Methyl salicylate ranked second among the volatile components in spring, summer, and autumn black tea. It has a fresh scent similar to wintergreen and mint and may be a major contributor to the fresh aroma quality of Rucheng Baimao black tea [54]. Previously published research indicates that a high content of methyl salicylate is associated with a high degree of freshness in Rucheng Baimao black tea, which is consistent with the results of this study [7]. It was also found that geraniol, linalool, methyl salicylate, and phenethyl alcohol were common among the top 10 volatile components in black tea from different seasons, suggesting that these volatile components play important roles in the aroma quality of Rucheng Baimao black tea in all three seasons. Geraniol and linalool are the most common alcohols in black tea and have long been identified as key aroma active components of black tea, they have distinct floral, fruity, and sweet aroma characteristics, which can significantly enhance the floral, fruity, and sweet aroma qualities of black tea [12,55]. Methyl salicylate and phenethyl alcohol are derived from amino acids and are recognized as key aroma substances in the world’s four famous black teas, the fresh and floral aroma characteristics they exhibit can significantly influence the formation of the corresponding qualities of black tea [12]. In conclusion, the dominant volatile components of Rucheng Baimao black tea vary in different seasons.

### 3.3. Analysis of Aroma Index of Black Tea in Different Seasons

To evaluate the differences in the aroma quality of Rucheng Baimao black tea in different seasons, this study calculated two flavor indices. Owuor’s flavor index (OFI) is the ratio of the sum of the contents of volatile components with sweet and floral aromas (Group Ⅱ) to the sum of the contents of volatile components with green/grassy notes (Group Ⅰ). The larger this ratio is, the higher the aroma quality [56]. The Wickremasinghe–Yamanishi ratio is the ratio of the sum of the contents of volatile components with a retention time shorter than that of linalool to the sum of the contents of volatile components with a retention time longer than that of linalool. The smaller this ratio is, the better the aroma quality [44]. In order to calculate the OFI index, the aroma characteristic descriptions of 140 volatile components were retrieved from the relevant literature and database websites and grouped, as shown in Table S1.

**Figure 3:**
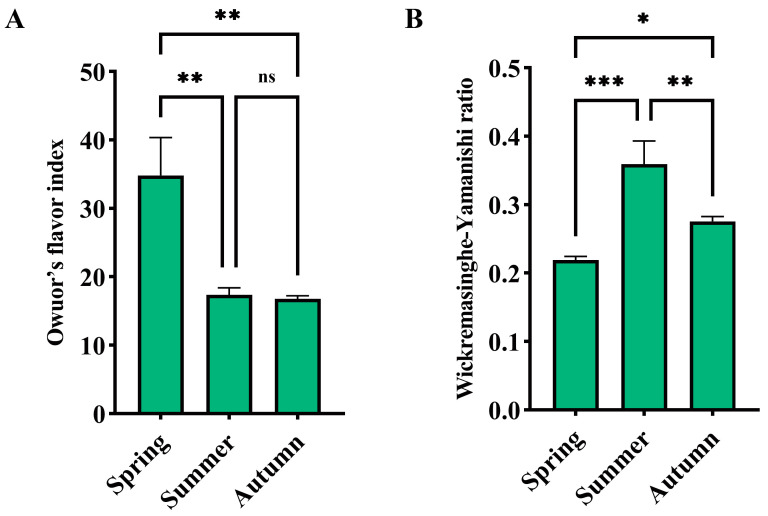
Flavor index of Rucheng Baimao black tea in different seasons. *, **, *** represent significant differences; ‘ns’ indicates no significant difference. (**A**) Owuor’s flavor index of black tea in different seasons; (**B**) wickremasinghe–yamanishi ratio of black tea in different seasons.

The calculation results of the flavor indices are shown in Figure 3. The OFI index of spring black tea was significantly higher than that of summer and autumn black tea, while there was no significant difference between summer and autumn (Figure 3A). Regarding the Wickremasinghe–Yamanishi ratio, the ratio of summer black tea was significantly higher than that of spring and autumn black tea, with spring black tea having the lowest ratio (Figure 3B). Considering the two flavor indices together, the aroma quality of spring black tea was the best, followed by autumn black tea, and summer black tea was the worst, which was consistent with the results of sensory evaluation (Table 1). Shi et al. believed that the aroma quality of tea produced in spring was the best, followed by autumn tea, and summer tea was the worst, which was consistent with the results of this study [57]. Huang et al.’s research showed that the aroma quality of autumn black tea was the best, followed by summer black tea, and spring black tea was the worst, which was contrary to the results of this study [58]. This might be related to differences in raw material varieties or processing techniques.

### 3.4. Multivariate Statistical Analysis

The abovementioned analysis results indicate that there are significant differences in the volatile components and aroma quality of Rucheng Baimao black tea in different seasons. To further explore the seasonal variations of the volatile components in Rucheng Baimao black tea, this study conducted a multivariate statistical analysis using the relative contents of 140 volatile components.

#### 3.4.1. Analysis of Differential Volatile Components in Black Tea from Different Seasons

**Figure 4:**
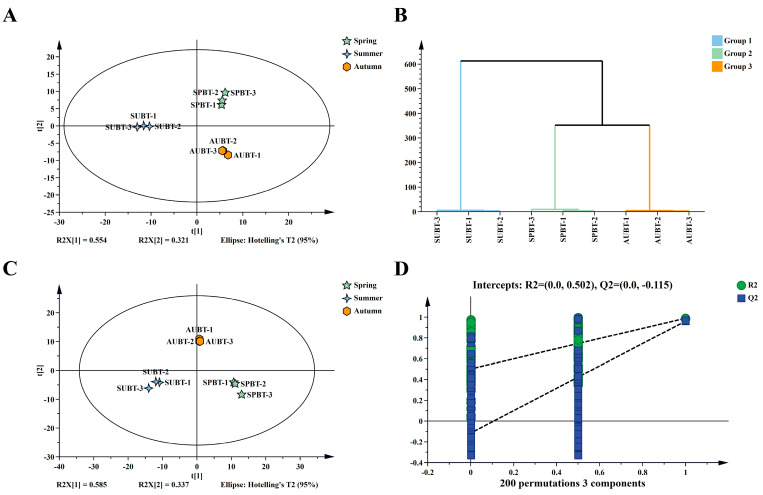
Multivariate statistical analysis of Rucheng Baimao black tea in different seasons. (**A**) Principal component analysis; (**B**) hierarchical cluster analysis; (**C**) partial least squares discriminant analysis; (**D**) permutation test.

First, an unsupervised PCA model was established using the relative contents of each volatile component. As shown in Figure 4A, the cumulative contribution rate of the first two principal components was 0.875. On the PCA score plot, the black tea samples from different seasons were clearly separated, indicating significant differences among samples from different seasons. Moreover, the three replicates within the same sample were relatively concentrated, suggesting good repeatability. Consistent results were obtained from HCA (Figure 4B).

To screen out the differential volatile components of Rucheng Baimao black tea in different seasons, a supervised PLS-DA model was further established. As shown in Figure 4C, for the PLS-DA model, R^2^X = 0.922 and R^2^Y = 0.993, indicating a good predictive effect of the model. Through 200 permutation tests, it was concluded that the model did not overfit (Q^2^ = −0.115), and it could be used to screen differential volatile components (Figure 4D).

Variable Importance in the Projection (VIP value) can be used to measure the contribution of a single variable to the PLS-DA model. Variables with VIP > 1 are generally considered to play an important role in the model discrimination. According to the principle of VIP > 1 and *p* < 0.05, the differential volatile components of Rucheng Baimao black tea in different seasons were screened. A total of 34 volatile components were selected, and the results are shown in Table 2. These volatile components can be used as potential differential volatile components to distinguish Rucheng Baimao black tea in spring, summer, and autumn.

**Figure 5:**
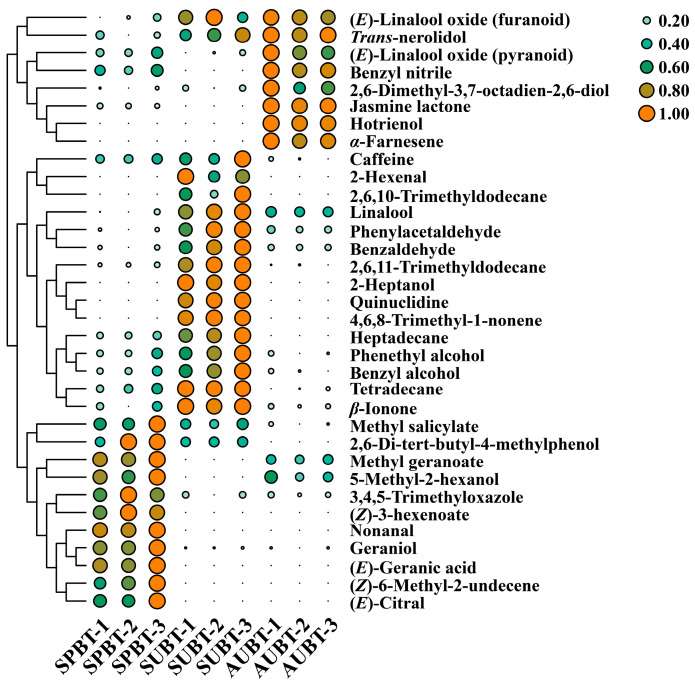
Heat map of differential volatile components in Rucheng Baimao black tea in different seasons.

To better visualize the distribution of the abovementioned differential volatile components among Rucheng Baimao black tea in different seasons, an HCA heatmap was drawn using the relative contents of the differential volatile components. As shown in Figure 5, the distribution of the 34 differential volatile components in black tea from spring, summer, and autumn showed a certain regularity and could be roughly divided into three groups. The first group included methyl salicylate, 2,6-di-tert-butyl-4-methylphenol, methyl geranoate, 5-methyl-2-hexanol, 3,4,5-trimethyloxazole, (*Z*)-3-hexenoate, nonanal, geraniol, (*E*)-geranic acid, (*Z*)-6-methyl-2-undecene, and (*E*)-citral, which accumulated the most in spring black tea. The second group consisted of caffeine, 2-hexenal, 2,6,10-Trimethyldodecane, linalool, phenylacetaldehyde, benzaldehyde, 2,6,11-trimethyldodecane, 2-heptanol, quinuclidine, 4,6,8-trimethyl-1-nonene, heptadecane, phenethyl alcohol, benzyl alcohol, tetradecane, and *β*-ionone, which accumulated the most in summer black tea. The third group contained (*E*)-linalool oxide (furanoid), *trans*-nerolidol, (*E*)-linalool oxide (pyranoid), benzyl nitrile, 2,6-dimethyl-3,7-octadien-2,6-diol, jasmine lactone, hotrienol, and *α*-farnesene, which accumulated the most in autumn black tea.

#### 3.4.2. rOAV Analysis of Differential Volatile Components

Not all volatile components in tea contribute to the aroma quality. Therefore, in this study, the relative odor activity value (rOAV) was calculated to evaluate the specific contributions of the abovementioned differential volatile components to the aroma of Rucheng Baimao black tea in different seasons. Generally, a volatile component with rOAV > 1 is considered to make an important contribution to the aroma quality [59]. By referring to the literature and databases, the odor thresholds of the differential volatile components were obtained and the rOAV values were calculated. The results are shown in Table 3. Among the 34 differential volatile components, the thresholds of 24 components were available for rOAV calculation. The rOAV values of 11 differential volatile components were greater than 1, including geraniol, linalool, methyl salicylate, nonanal, phenylacetaldehyde, benzaldehyde, phenethyl alcohol, benzyl alcohol, *β*-ionone, *trans*-nerolidol, and (*E*)-citral. Most of these volatile components exhibit floral, fruity, sweet, and fresh scents, jointly forming the aroma quality characteristics of Rucheng Baimao black tea. The rOAV values of phenethyl alcohol and *β*-ionone both exceeded 200, indicating that they may play an extremely important role in the formation of the floral aroma quality of Rucheng Baimao black tea. In addition, the aroma quality of tea is the result of the combined effect of volatile components in tea at different concentrations on the human olfactory nerve [60]. Therefore, volatile components with rOAV < 1 also contribute to the aroma quality of tea. Among the differential volatile components, the rOAV values of hotrienol, *α*-farnesene, 2-heptanol, (*E*)-linalool oxide (pyranoid), and 2-hexenal were between 0.1 and 1, suggesting that they may modify the aroma quality of Rucheng Baimao black tea. The rOAV values of caffeine, tetradecane, (*E*)-linalool oxide (furanoid), jasmine lactone, 5-methyl-2-hexanol, benzyl nitrile, and 2,6-di-tert-butyl-4-methylphenol were less than 0.1, which may have potential effects on the aroma quality of Rucheng Baimao black tea [59].

#### 3.4.3. Analysis of Key Differential Volatile Components

Based on the principles of VIP > 1, *p* < 0.05, and rOAV > 1, the abovementioned 11 differential volatile components with rOAV > 1 were selected as the key differential volatile components of Rucheng Baimao black tea in different seasons. It is speculated that the differences in the distribution of these key differential volatile components in Rucheng Baimao black tea in different seasons play an important role in the formation of the aroma quality of black tea in the corresponding seasons. To better analyze the impact of seasons on the key differential volatile components, this section will specifically analyze the distribution of their relative contents in Rucheng Baimao black tea in spring, summer, and autumn.

**Figure 6:**
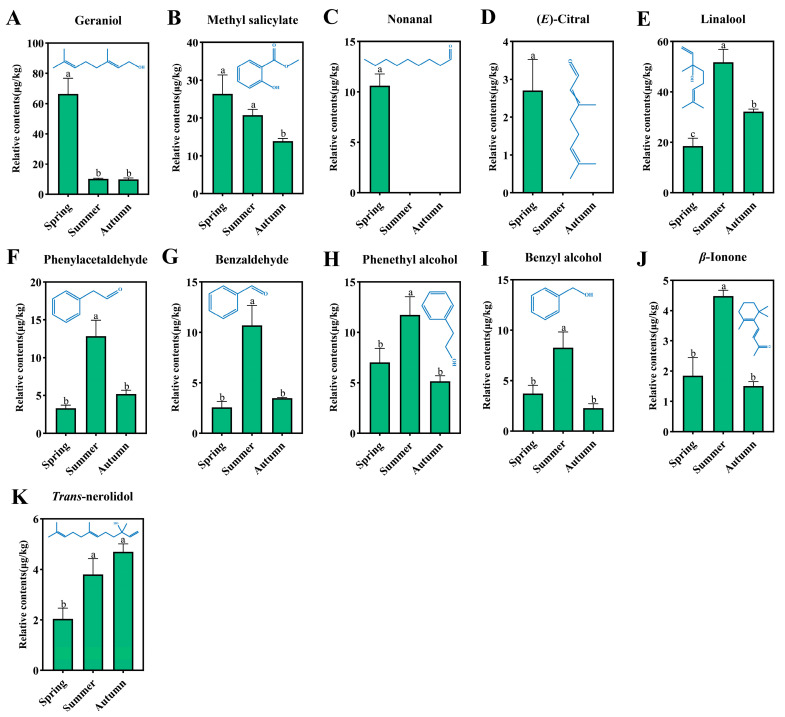
Comparison of key differential volatile components in Rucheng Baimao black tea across different seasons. (**A**) Comparison of the relative contents of geraniol in black tea in different seasons; (**B**) comparison of the relative contents of methyl salicylate in black tea in different seasons; (**C**) comparison of the relative contents of nonanal in black tea in different seasons; (**D**) comparison of the relative contents of (*E*)-citral in black tea in different seasons; (**E**) comparison of the relative contents of linalool in black tea in different seasons; (**F**) comparison of the relative contents of phenylacetaldehyde in black tea in different seasons; (**G**) comparison of the relative contents of benzaldehyde in black tea in different seasons; (**H**) comparison of the relative contents of phenethyl alcohol in black tea in different seasons; (**I**) comparison of the relative contents of benzyl alcohol in black tea in different seasons; (**J**) comparison of the relative contents of *β*-ionone in black tea in different seasons; (**K**) comparison of the relative contents of *trans*-nerolidol in black tea in different seasons. Different lowercase letters represent significant differences (*p* < 0.05).

As shown in Figure 6, the relative contents of geraniol, methyl salicylate, nonanal, and (*E*)-citral in spring black tea were significantly higher than those in summer and autumn (Figure 6A–D). Geraniol can be formed by the hydrolysis of glycosidic aroma precursors during tea processing [54]. Zhang et al. proposed that monoterpene alcohol glycosides, especially geraniol glycosides, have a high content in spring tea [66]. This may be the reason for the high relative content of geraniol in Rucheng Baimao spring black tea. Methyl salicylate exhibits a fresh and minty aroma, and nonanal shows a floral and fresh aroma. Moreover, methyl salicylate was considered to be related to the fresh aroma quality of Rucheng Baimao black tea in previous studies [7]. This implies that the high contents of methyl salicylate and nonanal in Rucheng Baimao spring black tea may contribute to the formation of a fresher aroma quality. (*E*)-citral has a fresh lemon-like aroma and has been identified as a key aroma component of black tea [67], which may also contribute to the formation of the freshness of Rucheng Baimao spring black tea.

In addition, the relative contents of linalool, phenylacetaldehyde, benzaldehyde, phenethyl alcohol, benzyl alcohol, and *β*-ionone in summer black tea were significantly higher than those in spring and autumn (Figure 6E–J). Except for the relative content of linalool in spring black tea being significantly lower than that in autumn black tea (Figure 6E), there were no significant differences in the remaining volatile components between spring and autumn black tea. Shi et al. proposed that spring tea contains more volatile components with fresh tea and floral aromas such as nonanal, linalool, and geraniol, while summer and autumn teas contain more floral components such as phenethyl alcohol and phenylacetaldehyde [57]. This is basically consistent with the results of this study. The difference is that in this study, summer black tea contains more linalool. Some studies have shown that lycopene can be oxidatively degraded into components such as linalool [60]. Lycopene belongs to carotenoids, which play a role as accessory pigments in the photosynthesis of tea plants and can be oxidatively degraded under factors such as light and high temperature [54]. It is speculated that the strong sunlight in summer promotes the oxidative degradation of lycopene, thus producing more linalool. This may be a reasonable explanation for this phenomenon. It is worth noting that Liu et al. research shows that the sharp increase in the content of linalool in autumn black tea is related to the increase in glycosidic aroma precursors with the growth of tea leaves [13], which is contrary to the results of this study. This may be related to differences in tea varieties, processing techniques, and detection methods. *β*-ionone can also be formed by the degradation of carotenoids. Its high relative content in summer black tea may also be related to the abovementioned reasons. The strong light and high temperature in summer promote the oxidative degradation of carotenoids into *β*-ionone.

The relative content of *trans*-nerolidol in summer and autumn black tea was significantly higher than that in spring (Figure 6K). It has a floral aroma characteristic. Generally, the climate in spring is cool, and the tea produced has a sharp and high-pitched aroma. With the increase in rainfall and temperature, the growth of tea plants accelerates, resulting in a decrease in the tea aroma. In autumn, the weather is clear and cool, and the temperature drops. The growth of tea plants slows down, and the aroma rebounds, forming the ‘autumn tea aroma’ [57]. Huang et al. research shows that the rich floral aroma of autumn tea is related to the relatively high contents of *trans*-nerolidol and others [68]. Therefore, it is speculated that the high relative content of *trans*-nerolidol in autumn black tea in this study may contribute to the formation of the floral aroma quality of Rucheng Baimao autumn black tea.

## 4. Conclusions

In this study, sensory evaluation and HS-SPME-GC-MS combined with rOAV were used to explore seasonal differences in the aroma quality and volatile components of Rucheng Baimao black tea. The results of sensory evaluation showed that black teas in spring, summer, and autumn all had floral aroma characteristics. The aroma quality of Rucheng Baimao spring black tea was the best, followed by autumn black tea, and summer black tea was the worst. The detection of volatile components revealed that a total of 140 volatile components were detected in black teas from different seasons. Among them, alcohols, esters, and alkanes were the main categories of volatile components. The overall trend of the relative content of volatile components was summer black tea > spring black tea > autumn black tea. The dominant volatile components in black teas of different seasons also varied. Geraniol had the highest relative content in spring black tea, linalool had the highest relative content in summer and autumn black teas, and methyl salicylate ranked second in black teas of all three seasons. The aroma index analysis indicated that the aroma quality of spring black tea was the best, followed by autumn black tea, and summer black tea was the worst, which confirmed the results of sensory evaluation. Using multivariate statistical analysis, 34 differential volatile components were screened out which could be used as potential substances to distinguish black teas of different seasons. By combining VIP > 1, *p* < 0.05, and rOAV > 1, 11 key differential volatile components were selected from black teas of different seasons. Geraniol, methyl salicylate, nonanal, and (*E*)-citral accumulated the most in spring black tea, linalool, phenylacetaldehyde, benzaldehyde, phenethyl alcohol, benzyl alcohol, and β-ionone accumulated the most in summer black tea, and *trans*-nerolidol accumulated the most in autumn black tea. The differences in their distribution in black teas of different seasons were the material basis for the seasonal differences in the aroma quality of Rucheng Baimao black tea. The results of this study are helpful for deepening the understanding of the aroma components of Rucheng Baimao black tea and provide a certain reference for the regulation of its aroma quality.

However, this study still has certain limitations. On the one hand, the influence of volatile components on the aroma quality of black tea is not merely singular. The interactions between volatile components can also have an impact on the aroma quality of black tea. On the other hand, the sensory evaluation of tea in this study mainly relies on human judgment. Although five individuals were selected in this study to reduce the subjective biases in sensory evaluation, there are still defects of being not completely objective. Based on this, in future research, it is imperative to utilize molecular sensory omics methods such as triangle tests, electronic noses, gas chromatography–olfactometry (GC-O), aroma recombination, and aroma omission to deeply explore the aroma quality of Rucheng Baimao black tea.

## Figures and Tables

**Table 1:** Sensory aroma evaluation of Rucheng Baimao black tea in different seasons.

Tea Samples	Seasons	Aroma	Score
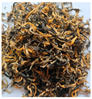	Spring	Floral–fruity aroma, refreshing sensation, with sweet fragrance	90.20 ± 1.17 a
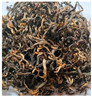	Summer	Possesses a floral aroma	82.40 ± 0.80 c
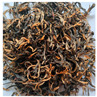	Autumn	The floral fragrance is pure and harmonious	86.60 ± 1.36 b

Note: Different lowercase letters indicate significant differences (*p* < 0.05).

**Table 2:** Differential volatile components of Rucheng Baimao black tea in different seasons.

Volatile Components	VIP Value	*p*-Value
Geraniol	4.70	0.00
Linalool	3.07	0.00
Hotrienol	2.48	0.00
Methyl salicylate	2.31	0.01
*α*-Farnesene	2.19	0.00
Nonanal	2.05	0.00
Quinuclidine	2.04	0.00
(*E*)-Geranic acid	1.95	0.00
Caffeine	1.74	0.02
Phenylacetaldehyde	1.71	0.00
Benzaldehyde	1.64	0.00
Methyl geranoate	1.60	0.00
Phenethyl alcohol	1.56	0.00
Tetradecane	1.52	0.00
2-Heptanol	1.49	0.00
Benzyl alcohol	1.49	0.00
(*Z*)-6-Methyl-2-undecene	1.46	0.00
(*E*)-Linalool oxide (furanoid)	1.39	0.01
4,6,8-Trimethyl-1-nonene	1.37	0.00
(*E*)-Linalool oxide (pyranoid)	1.16	0.00
3,4,5-Trimethyloxazole	1.16	0.00
Jasmine lactone	1.15	0.00
5-Methyl-2-hexanol	1.15	0.00
2,6,11-Trimethyldodecane	1.14	0.00
2,6-Dimethyl-3,7-octadien-2,6-diol	1.13	0.00
2-Hexenal	1.11	0.00
Benzyl nitrile	1.08	0.00
2,6,10-Trimethyldodecane	1.06	0.03
(*Z*)-3-Hexenoate	1.05	0.00
2,6-Di-tert-butyl-4-methylphenol	1.05	0.01
*β*-Ionone	1.05	0.00
*Trans*-nerolidol	1.04	0.00
(*E*)-Citral	1.02	0.00
Heptadecane	1.00	0.00

**Table 3:** ROAV analysis of differential volatile components in Rucheng Baimao black tea across different seasons.

Volatile Components	Thresholds (μg/kg)	rOAV		
		SPBT	SUBT	AUBT
Geraniol	1	66.34 ± 8.50	10.25 ± 0.36	9.99 ± 0.71
Linalool	6	3.08 ± 0.43	8.64 ± 0.68	5.37 ± 0.13
Hotrienol	110	-	-	0.10 ± 0.00
Methyl salicylate	16	1.65 ± 0.26	1.30 ± 0.08	0.87 ± 0.04
α-Farnesene	87	-	-	0.10 ± 0.01
Nonanal	1	10.63 ± 0.94	-	-
Quinuclidine	-	-	-	-
(*E*)-Geranic acid	-	-	-	-
Caffeine	2000	<0.1	<0.1	<0.1
Phenylacetaldehyde	0.3	11.09 ± 1.07	42.87 ± 5.68	17.30 ± 1.30
Benzaldehyde	3	0.85 ± 0.16	3.56 ± 0.54	1.15 ± 0.02
Methyl geranoate	-	-	-	-
Phenethyl alcohol	0.015	469.02 ± 74.84	781.15 ± 98.84	343.60 ± 29.48
Tetradecane	1000	<0.1	<0.1	<0.1
2-Heptanol	13	-	0.45 ± 0.02	-
Benzyl alcohol	2.54	1.46 ± 0.27	3.25 ± 0.50	0.90 ± 0.14
(*Z*)-6-Methyl-2-undecene	-	-	-	-
(*E*)-Linalool oxide (furanoid)	320	<0.1	<0.1	<0.1
4,6,8-Trimethyl-1-nonene	-	-	-	-
(*E*)-Linalool oxide (pyranoid)	50	<0.1	<0.1	0.11 ± 0.01
3,4,5-Trimethyloxazole	-	-	-	-
Jasmine lactone	2000	<0.1	-	<0.1
5-Methyl-2-hexanol	330	<0.1	-	<0.1
2,6,11-Trimethyldodecane	-	-	-	-
2,6-Dimethyl-3,7-octadien-2,6-diol	-	-	-	-
2-Hexenal	30	-	0.12 ± 0.03	-
Benzyl nitrile	1000	<0.1	-	<0.1
2,6,10-Trimethyldodecane	-	-	-	-
(*Z*)-3-Hexenoate	-	-	-	-
2,6-Di-tert-butyl-4-methylphenol	1000	<0.1	<0.1	-
*β*-Ionone	0.007	263.75 ± 70.63	639.95 ± 22.18	215.70 ± 16.87
*Trans*-nerolidol	0.25	8.18 ± 1.40	15.23 ± 2.06	18.83 ± 1.01
(*E*)-Citral	0.5	5.41 ± 1.34	-	-
Heptadecane	10,000,000	<0.1	<0.1	-

Note: The threshold refers to that of the compound in water; the threshold values are sourced from the VCF website (https://www.vcf-online.nl/VcfHome.cfm (accessed on 15 January 2025)) and the relevant literature [39,40,50,52,61,62,63,64,65].

## Data Availability

The original contributions presented in this study are included in the article/Supplementary Materials. Further inquiries can be directed to the corresponding authors.

## Supplementary Materials

The following supporting information can be downloaded at https://www.mdpi.com/article/10.3390/foods14050763/s1, Table S1: Volatile components of Rucheng Baimao black tea in different seasons.

## References

[1] Wu H.-L., Huang W.-J., Chen Z.-J., Chen Z., Shi J.-F., Kong Q., Sun S.-L., Jiang X.-H., Chen D., Yan S.-J. (2019). GC–MS-based metabolomic study reveals dynamic changes of chemical compositions during black tea processing. Food Res. Int..

[2] Yang Z.-Y., Baldermann S., Watanabe N. (2013). Recent studies of the volatile compounds in tea. Food Res. Int..

[3] Li S.-M., Lo C.-Y., Pan M.-H., Lai C.-S., Ho C.-T. (2013). Black tea: Chemical analysis and stability. Food Funct..

[4] Yin X., Huang J.-A., Huang J., Wu W.-L., Tong T., Liu S.-J., Zhou L.-Y., Liu Z.-H., Zhang S.-G. (2022). Identification of volatile and odor-active compounds in Hunan black tea by SPME/GC-MS and multivariate analysis. LWT.

[5] Yin X., Zhang S.-G., Huang J., Bao X.-C., Zhou L.-Y., Dai W.-D., Zhao C.-J., Huang J.-A., Liu Z.-H. (2019). Study on the chemical constituents of Hunan black tea. J. Tea Sci..

[6] Li N., Zhong X.-G., Huang H.-S., Chen Y.-Y., Su B.-W. (2019). Research progress on characteristic tea tree resources of Rucheng Baimao tea. J. Tea Commun..

[7] Ouyang J., Jiang R.-G., Chen H.-Y., Liu Q., Yi X.-Q., Wen S., Huang F.-F., Zhang X.-Y., Li J., Wen H.-T. (2024). Characterization of key odorants in ‘Baimaocha’ black teas from different regions. Food Chem. X.

[8] Zhong X.-G., Huang H.-S., Li N., Su B.-W. (2022). Effect of withering and fermentation technology on the quality of “Rucheng Baimaocha” processed black tea. Food Ferment. Ind..

[9] Kumazawa K., Wada Y., Masuda H. (2006). Characterization of epoxydecenal isomers as potent odorants in black tea (Dimbula) infusion. J. Agric. Food Chem..

[10] Chen Q.-C., Zhu Y., Liu Y.-F., Liu Y., Dong C.-W., Lin Z., Teng J. (2022). Black tea aroma formation during the fermentation period. Food Chem..

[11] Joshi R., Gulati A. (2015). Fractionation and identification of minor and aroma-active constituents in Kangra orthodox black tea. Food Chem..

[12] Kang S.-Y., Yan H., Zhu Y., Liu X., Lv H.-P., Zhang Y., Dai W.-D., Guo L., Tan J.-F., Peng Q.-H. (2019). Identification and quantification of key odorants in the world’s four most famous black teas. Food Res. Int..

[13] Liu H.-C., Xu Y.-J., Wu J.-J., Wen J., Yu Y.-S., An K.-J., Zou B. (2021). GC-IMS and olfactometry analysis on the tea aroma of Yingde black teas harvested in different seasons. Food Res. Int..

[14] Wang J., Li X.-H., Wu Y., Qu F.-F., Liu L., Wang B.-Y., Wang P.-Q., Zhang X.-F. (2022). HS−SPME/GC−MS reveals the season effects on volatile compounds of green tea in high−latitude region. Foods.

[15] Huang H.-S., Su B.-W., Zhong X.-G., Zhao X., Yin X., Huang H. (2016). A comparative analysis of the quality of time black tea with several special resources (varieties) in Hunan. J. Tea Commun..

[16] Wen H.-T., Xie S.-L., Li T.-Z., He Z.-H. (2019). Development status and reflections on the tea industry in Rucheng County, Hunan province. China Tea.

[17] (2018). Methodology of Sensory Evaluation of Tea.

[18] Zhu J.-Y., Li Y., Shi L.-L., Zhou Y.-B., Wen H.-T. (2025). Dynamic changes in aroma components during the processing of Rucheng Baimao (*Camellia pubescens*) black tea. Mod. Food Sci. Technol..

[19] Chen H.-Y., Zhang X.-M., Jiang R.-G., Ouyang J., Liu Q., Li J., Wen H.-T., Li Q., Chen J.-H., Xiong L.-G. (2023). Characterization of aroma differences on three drying treatments in Rucheng Baimao (*Camellia pubescens*) white tea. LWT.

[20] Jiang R.-G., Huang Y., Jin Y.-L., Li Y.-D., Huang J.-A., Li Q. (2021). Study of aroma compounds and their source in Fu brick tea. J. Food Sci. Biotechnol..

[21] Xiao Z.-B., Wu Q.-Y., Niu Y.-W., Wu M.-L., Zhu J.-C., Zhou X., Chen X.-M., Wang H.-L., Li J., Kong J.-L. (2017). Characterization of the key aroma compounds in five varieties of mandarins by gas chromatography–olfactometry, odor activity values, aroma recombination, and omission analysis. J. Agric. Food Chem..

[22] Chen W., Hu D., Miao A.-Q., Qiu G.-J., Qiao X.-Y., Xia H.-L., Ma C.-Y. (2022). Understanding the aroma diversity of Dancong tea (*Camellia sinensis*) from the floral and honey odors: Relationship between volatile compounds and sensory characteristics by chemometrics. Food Control.

[23] Fang X., Xu W.-C., Jiang G.-X., Sui M.-Y., Xiao J.-Y., Ning Y.-Y., Niaz R., Wu D.-W., Feng X.-G., Chen J.-H. (2024). Monitoring the dynamic changes in aroma during the whole processing of Qingzhuan tea at an industrial scale: From fresh leaves to finished tea. Food Chem..

[24] Fu Z.-J., Hao S.-X., Zhou J.-Y., Feng W.-J., Zhu M.-Y., Wu J.-L., Zhang Y.-Z., Yu Y. (2024). Profiling volatile compounds in fresh leaves of 22 major oolong tea germplasm cultivated in Fujian of China. Sci. Hortic..

[25] He C., Li Y.-C., Zhou J.-T., Yu X.-L., Zhang D., Chen Y.-Q., Ni D.-J., Yu Z. (2022). Study on the suitability of tea cultivars for processing oolong tea from the perspective of aroma based on olfactory sensory, electronic nose, and GC-MS data correlation analysis. Foods.

[26] Huang C.-X., Kun J.-R., Lin Y.-Z., Dai H.-W., Weng T.-J., Li Z.-W., Tong H.-R. (2024). Analysis of aroma quality of pearl orchid at different flowering stages and Zhulan scented tea. Food Sci..

[27] Huang W.-J., Fang S.-M., Wang J., Zhuo C., Luo Y.-H., Yu Y.-L., Li L.-Q., Wang Y.-J., Deng W.-W., Ning J.-M. (2022). Sensomics analysis of the effect of the withering method on the aroma components of Keemun black tea. Food Chem..

[28] Lin Y.-H., Wei R., Zheng J.J., Zheng J., Zhang W., Yasir M., Kayama K., Liu X.-Q., Su Z.-C. (2024). Volatile and non-volatile compounds profiling and their role in sensory and antioxidative attributes of two species of “red snow tea” (Lethariella). J. Food Compos. Anal..

[29] Ren W.-W., Xu M.-T., Chen W.-X., Gu M.-Y., Zeng S.-S., Jin S., Chen B.-W., Ye N.-X. (2024). Analysis of aroma components in Anxi Huang Jingui oolong tea tea using different wrapping-twisting methods via HS-SPME-GC-MS. Sci. Technol. Food Ind..

[30] Shen S.-S., Wu H.-T., Li T.-H., Sun H.-R., Wang Y.-J., Ning J.-M. (2023). Formation of aroma characteristics driven by volatile components during long-term storage of An tea. Food Chem..

[31] Sun Z.-G., Yang X.-D., Wu W., Yang P.-F., Yang J., Bai B., Huang K., Liu Y.-Z., Liu S., Cui F.-X. (2024). Characteristic aroma components of cucumber based on odor activity value analysis before and after rotary spinning cone column treatment. Food Ferment. Ind..

[32] Wen S., Sun L.-L., Zhang S.-W., Chen Z.-Z., Chen R.-H., Li Z.-G., Lai X.-F., Zhang Z.-B., Cao J.-X., Li Q. (2023). The formation mechanism of aroma quality of green and yellow teas based on GC-MS/MS metabolomics. Food Res. Int..

[33] Xiao Z.-B., Wang H.-L., Niu Y.-W., Zhu J.-C., Ma N. (2018). Analysis of aroma components in four chinese Congou black teas by odor active values and aroma extract dilution analysis coupled with partial least squares regression. Food Sci..

[34] Xu Y.-J., Zhou H.-C., Zhang X.-L., Liu Y.-Q., Lei P.-D. (2024). Differences in flavor quality and chemical composition of 1-year-stored Keemun black tea. Food Ferment. Ind..

[35] Yun J., Cui C.-J., Zhang S.-H., Zhu J.-J., Peng C.-Y., Cai H.-M., Yang X.-G., Hou R.-Y. (2021). Use of headspace GC/MS combined with chemometric analysis to identify the geographic origins of black tea. Food Chem..

[36] Zhu Y., Lv H.-P., Shao C.-Y., Kang S., Zhang Y., Guo L., Dai W.-D., Tan J.-F., Peng Q.-H., Lin Z. (2018). Identification of key odorants responsible for chestnut-like aroma quality of green teas. Food Res. Int..

[37] Cao B.-Y., Pu D.-D., Zheng R.-Y., Meng R.-X., Sun B.-G., Zhang Y.-Y. (2023). Characterization of the aroma-active compounds among 20 pungent spices by SAFE-SPME-GC-MS/O. Food Sci..

[38] Ge C., Zhang P., Li J.-K., Jia X.-Y., Wu D., Li C.-Y., Liu L. (2025). Storage resistance and volatile matter changes in two varieties of raspberries. Sci. Technol. Food Ind..

[39] Guo X.-Y., Ho C.-T., Schwab W., Wan X.-C. (2021). Aroma profiles of green tea made with fresh tea leaves plucked in summer. Food Chem..

[40] Guo X.-Y., Ho C.-T., Schwab W., Wan X.-C. (2021). Effect of the roasting degree on flavor quality of large-leaf yellow tea. Food Chem..

[41] Guo X.-Y., Ho C.-T., Wan X.-C., Zhu H., Liu Q., Wen Z. (2021). Changes of volatile compounds and odor profiles in Wuyi rock tea during processing. Food Chem..

[42] Guo X.-Y., Schwab W., Ho C.-T., Song C.-K., Wan X.-C. (2022). Characterization of the aroma profiles of oolong tea made from three tea cultivars by both GC–MS and GC-IMS. Food Chem..

[43] Miao Y.-W., Zhou J.-Y., Yang C.-M., Luo Z.-F., Wang Y., Gong Z.-L., Tong H.-R. (2024). Analysis of quality in Shoumei white tea from different tea plant varieties. Sci. Technol. Food Ind..

[44] Qiao D.-H., Mi X.-Z., An Y.-L., Xie H., Cao K.-M., Chen H.-R., Chen M.-Y., Liu S.-R., Chen J., Wei C.-L. (2021). Integrated metabolic phenotypes and gene expression profiles revealed the effect of spreading on aroma volatiles formation in postharvest leaves of green tea. Food Res. Int..

[45] Qiu D.-Y., Zhu C.-Y., Fan R.-Y., Mao G.-L., Huang Y.-J., Wu P.-Z., Zeng J.-W. (2024). Analysis of the change of major aroma volatile compounds of ‘Gonggan’ mandarin fruits during postharvest storage. Food Ferment. Ind..

[46] Shi Y.-L., Zhu Y., Ma W.-J., Yang G.-Z., Wang M.-Q., Shi J., Peng Q.-H., Lin Z., Lv H.-P. (2021). Research progress on the volatile compounds of premium roasted green tea. J. Tea Sci..

[47] Wang Z.-X., Su D., Ren H.-T., Lv Q., Ren L., Li Y.-L., Zhou H.-J. (2022). Effect of different drying methods after fermentation on the aroma of Pu-erh tea (ripe tea). LWT.

[48] Wu Q.-J., Zhou Z., Lin F.-M., Qi S.-Y., Zhou M.-S., Peng L.-Q., Sun W.-J. (2024). Quality difference and its forming material basis of Wuyi Shuixian rock Tea with different management methods. Chin. J. Trop. Crops.

[49] Xu M.-T., Gu M.-Y., Chen J., Wei M.-X., Chen Q., Wu W.-X., Zheng Y.-C., Ye N.-X. (2024). Flavor quality analysis of ‘Jinmudan’ and ‘Jinguanyin’ high aroma black tea using metabolomics. Food Sci..

[50] Yang G.-Z., Zhou M.-X., Shi J., Peng Q.-H., Lin Z., Lv H.-P., Simal-Gandara J. (2023). How anaerobic treatment is controlling the volatile components and key odorants of purple-colored leaf tea. J. Food Compos. Anal..

[51] Yin X., Xiao Y.-B., Wang K.-F., Wu W.-L., Huang J., Liu S.-J., Zhang S.-G. (2023). Effect of shaking manners on floral aroma quality and identification of key floral-aroma-active compounds in Hunan black tea. Food Res. Int..

[52] Zhou J.-T., He C., Qin M.-X., Luo Q.-Q., Jiang X.-F., Zhu J.-Y., Qiu L., Yu Z., Zhang D., Chen Y.-Q. (2023). Characterizing and decoding the effects of different fermentation levels on key aroma substances of Congou black tea by sensomics. J. Agric. Food Chem..

[53] Dudareva N., Klempien A., Muhlemann J.K., Kaplan I. (2013). Biosynthesis, function and metabolic engineering of plant volatile organic compounds. New Phytol..

[54] Ho C.-T., Zheng X., Li S.-M. (2015). Tea aroma formation. Food Sci. Hum. Wellness.

[55] Zhai X.-T., Zhang L., Granvogl M., Ho C.-T., Wan X.-C. (2022). Flavor of tea (*Camellia sinensis*): A review on odorants and analytical techniques. Compr. Rev. Food Sci. Food Saf..

[56] Owuor P.O. (1992). Comparison of gas chromatographic volatile profiling methods for assessing the flavour quality of kenyan black teas. J. Sci. Food Agric..

[57] Shi Z.-P. (2010). Tea Evaluation and Inspection.

[58] Huang H., Yu P.-H., Zhao X., Zhong N., Zheng H.-F. (2020). HS-SPME-GC-MS analysis of volatile components of Congou black tea processed from Baojing Huangjincha 1 from different harvesting seasons. Food Sci..

[59] Su D., He J.-J., Zhou Y.-Z., Li Y.-L., Zhou H.-J. (2022). Aroma effects of key volatile compounds in Keemun black tea at different grades: HS-SPME-GC-MS, sensory evaluation, and chemometrics. Food Chem..

[60] Wan X.-C. (2003). Tea Biochemistry.

[61] Cui J.-L., Zhai X.-T., Guo D.-Y., Du W.-K., Gao T., Zhou J., Schwab W.G., Song C.-K. (2022). Characterization of key odorants in Xinyang Maojian green tea and their changes during the manufacturing process. J. Agric. Food Chem..

[62] Gemert L.J.v. (2018). Compilations of Odour Threshold Values in Air, Water and Other Media.

[63] Huang J.-F., Yan T.-Y., Yang J.-F., Xu H. (2023). Aroma components analysis and origin differentiation of black tea based on ATD-GC-MS and E-nose. Horticulturae.

[64] Liu C., Wang C., Zheng T.-T., Zhao M.-M., Gong W.-Y., Wang Q.-M., Yan L., Zhang W.-J. (2022). Characterization of key odor-active compounds in sun-dried black tea by sensory and instrumental-directed flavor analysis. Foods.

[65] Yang Y.-Q., Zhu H.-K., Chen J.-Y., Xie J.-L., Shen S., Deng Y.-L., Zhu J.-Y., Yuan H.-B., Jiang Y.-W. (2022). Characterization of the key aroma compounds in black teas with different aroma types by using gas chromatography electronic nose, gas chromatography-ion mobility spectrometry, and odor activity value analysis. LWT.

[66] Zhang Z.-Z., Wan X.-C., Shi Z.-P., Xia T. (2003). Studies on the content of glycosidic tea aroma precursors in leaves of Zhuye during different seasons green tea processing and storage. Food Ferment. Ind..

[67] Qin X.-X., Zhou J.-T., He C., Qiu L., Zhang D., Yu Z., Wang Y., Ni D.-J., Chen Y.-Q. (2023). Non-targeted metabolomics characterization of flavor formation of Lichuan black tea processed from different cultivars in Enshi. Food Chem. X.

[68] Huang H.-Q., Yang Y., Liu Z.-Z., Fang Z., Lin J.-Q., Zhan S.-X., Zhan X.-Y., Chen C.-S., Sun Y. (2023). Quality analysis of a new tea line ‘606’ oolong tea in different seasons. Sci. Technol. Food Ind..

