# Supplementary material for: Analysis of Differences in Volatile Components of Rucheng Baimao (Camellia pubescens) Black Tea in Different Seasons
Source: Foods. 2025 Feb 24;14(5):763. doi: 10.3390/foods14050763 (PMC11899084; doi:10.3390/foods14050763)
Supplement: Supplementary file 1 [file foods-14-00763-s001.zip › foods-3467840-supplementary.pdf]

Table S1 Volatile components of Rucheng Baimao black tea in different seasons

Volatile Components	CAS	Category	Retention Time	Retention Index	Reference Retention Index	Identification	Relative Content（μg/kg）			Aroma	OFI Group
							SPBT	SUBT	AUBT		
2-Hexenal	505-57-7	Aldehydes	9.242	851.15	847	MS,RI	-	3.47±1.02	-	Fat, rancid	-
(*Z*)-6-Methyl-2-undecene	74630-43-6	Alkenes	9.257	851.54	-	MS	5.59±1.59	-	-	-	-
2-Heptanol	543-49-7	Alcohols	11.1	900.1	900	MS,RI	-	5.90±0.29	-	Citrus-like	-
5-Methyl-2-hexanol	627-59-8	Alcohols	11.149	901.12	-	MS	4.87±0.89	-	2.45±0.89	-	-
Benzaldehyde	100-52-7	Aldehydes	14.121	962.82	961	MS,RI	2.56±0.48	10.69±1.62	3.46±0.05	Almond, fruity, sweet	II
β-Myrcene	123-35-3	Alkenes	15.425	989.89	991	MS,RI	2.06±0.56	-	-	Fruity, herbal	I
2-Amylfuran	3777-69-3	Others	15.451	990.43	993	MS,RI	-	2.05±0.35	-	Green bean, butter	I
(*Z*)-3-hexenoate	64187-83-3	Esters	16	1001.72	-	MS	2.79±0.43	-	-	Fruity	-
(*E*,*E*)-2,4-Heptadienal	4313-03-5	Aldehydes	16.527	1012.02	1019	MS,RI	-	2.26±0.12	-	Fatty, grassy	I
3,4,5-Trimethyloxazole	10557-82-1	Others	16.774	1016.85	-	MS	6.67±0.73	3.23±0.39	3.18±0.20	-	-
Benzyl alcohol	100-51-6	Alcohols	17.656	1034.09	1031	MS,RI	3.72±0.68	8.27±1.27	2.28±0.35	Floral, fruity	II
Phenylacetaldehyde	122-78-1	Aldehydes	18.264	1045.97	1040	MS,RI	3.33±0.32	12.86±1.70	5.19±0.39	Floral, fruity, sweet	II
4-Methylheptadecane	26429-11-8	Alkanes	18.983	1060.03	-	MS	-	0.95±0.03	-	-	-
(*E*)-Linalool oxide (furanoid)	34995-77-2	Alcohols	19.585	1071.79	1093	MS,RI	4.77±0.68	9.23±1.73	10.07±0.44	Floral, citrus-like	II
Heptanoic acid	111-14-8	Others	19.938	1078.69	1073	MS,RI	2.26±0.35	-	-	Floral, sour	II
(*Z*)-Linalool oxide (furanoid)	5989-33-3	Alcohols	20.405	1087.82	1088	MS,RI	4.64±0.50	2.85±0.27	4.78±0.13	Floral, dust	II
Linalool	78-70-6	Alcohols	21.096	1101.35	1102	MS,RI	18.51±2.57	51.83±4.11	32.22±0.80	Floral, grape-like	II
Quinuclidine	100-76-5	Others	21.268	1104.78	-	MS	-	11.13±0.74	-	-	-
Hotrienol	29957-43-5	Alcohols	21.272	1104.86	1106	MS,RI	-	-	10.79±0.55	Fresh, floral, fruity	II
Nonanal	124-19-6	Aldehydes	21.277	1104.96	1106	MS,RI	10.63±0.94	-	-	Floral, fresh	II
Phenethyl alcohol	60-12-8	Alcohols	21.736	1114.1	1109	MS,RI	7.04±1.12	11.72±1.48	5.15±0.44	Floral, fruity, sweet, rose-like	II
Benzyl nitrile	140-29-4	Others	23.005	1139.36	1154	MS,RI	1.15±0.34	-	2.62±0.23	-	-
(*E*)-3-nonen-1-ol	10339-61-4	Alcohols	23.764	1154.47	-	MS	-	0.72±0.19	-	Fresh, waxy, green, mushroom-like	I
(*E*)-2-Nonenal	18829-56-6	Aldehydes	24.079	1160.74	1163	MS,RI	0.56±0.09	1.00±0.30	0.69±0.12	Cucumber, grassy	I
(*Z*)-Linalool oxide (pyranoid)	14049-11-7	Alcohols	24.627	1171.65	1175	MS,RI	2.85±0.19	3.52±0.39	3.66±0.46	Floral	II
(*E*)-Linalool oxide (pyranoid)	39028-58-5	Alcohols	24.844	1175.97	1162	MS,RI	3.44±0.47	2.42±0.24	5.36±0.59	Floral, sweet	II
Terpinen-4-ol	20126-76-5	Alcohols	25.274	1184.53	1182	MS,RI	-	0.75±0.00	-	Woody, herbal, pepper	I
2,6-Dimethyl-3,7-octadien-2,6-diol	13741-21-4	Alcohols	25.493	1188.89	1189	MS,RI	1.30±0.08	1.56±0.24	3.82±0.76	Sweet, fresh	II
1-Dodecene	112-41-4	Alkenes	25.648	1191.98	1189	MS,RI	-	0.28±0.00	-	Floral, waxy	II
1-Dodecanol	112-53-8	Alcohols	25.671	1192.43	-	MS	-	-	0.36±0.05	Honey, coconut	II
Methyl salicylate	119-36-8	Esters	25.801	1195.02	1190	MS,RI	26.37±4.10	20.74±1.30	13.87±0.60	Fresh, minty	II
α-Terpineol	98-55-5	Alcohols	25.962	1198.23	1198	MS,RI	-	0.25±0.08	0.14±0.05	Pine, lily-like, fruity	II
Dodecane	112-40-3	Alkanes	26.042	1199.82	1200	MS,RI	0.37±0.00	2.80±0.11	0.59±0.11	Alkane-like	-
Decanal	112-31-2	Aldehydes	26.372	1206.7	1208	MS,RI	0.28±0.13	0.66±0.18	0.60±0.14	Floral, citrus-like	II
(*E*,*E*)-Farnesol	106-28-5	Alcohols	26.893	1217.59	-	MS	-	0.87±0.22	-	Floral, sweet, lily	II
β-Cyclocitral	432-25-7	Aldehydes	27.1	1221.91	1217	MS,RI	0.41±0.08	1.37±0.24	0.40±0.07	Floral and fruity	II
Nerol	106-25-2	Alcohols	27.251	1225.06	1226	MS,RI	1.52±0.21	1.06±0.16	0.52±0.04	Floral, fruity, sweet	II
Cis-3-Hexenyl 2-Methylbutanoate	53398-85-9	Esters	27.479	1229.82	1234	MS,RI	0.88±0.25	0.74±0.12	1.14±0.04	Green, fresh	I

Hexyl 2-Methylbutyrate	10032-15-2	Esters	27.747	1235.42	1235	MS,RI	-	-	1.35±0.12	Green, waxy, fruity	-
Cyclohexyl pentanoate	1551-43-5	Esters	27.771	1235.92	-	MS	-	0.66±0.03	-	-	-
(*Z*)-Citral	106-26-3	Aldehydes	27.93	1239.24	1239	MS,RI	0.71±0.07	-	-	Lemon-like, fruity, sweet	II
Geraniol	106-24-1	Alcohols	28.604	1253.32	1249	MS,RI	66.34±8.5	10.25±0.36	9.99±0.71	Floral, fruity	II
beta-Homocyclocitral	472-66-2	Aldehydes	28.886	1259.21	1251	MS,RI	-	0.55±0.14	-	-	-
Trans-2-Decenal	3913-81-3	Aldehydes	29.098	1263.64	1268	MS,RI	-	-	0.43±0.08	Fatty, hay, fishy	I
(*E*)-Citral	5392-40-5	Aldehydes	29.331	1268.5	1264	MS,RI	2.71±0.67	-	-	Lemon-like, sweet	II
2-Phenyl-2-butenal	4411-89-6	Aldehydes	29.484	1271.7	1279	MS,RI	-	0.74±0.13	-	Sweet	II
2,6,11-Trimethyldodecane	31295-56-4	Alkanes	29.597	1274.06	1275	MS,RI	0.65±0.10	3.81±0.33	0.34±0.02	-	-
5-Butylnonane	17312-63-9	Alkanes	30.014	1282.77	-	MS	-	1.01±0.25	-	-	-
5-Methyl-5-propylnonane	17312-75-3	Alkanes	30.018	1282.85	-	MS	-	1.22±0.57	-	-	-
2,6,10-Trimethyldodecane	3891-98-3	Alkanes	30.28	1288.32	-	MS	-	3.45±1.83	-	-	-
Ethyl nonanoate	123-29-5	Esters	30.558	1294.13	1296	MS,RI	0.83±0.16	-	-	Fruity	-
2-Butyl-1-octanol	3913-02-8	Alcohols	30.825	1299.71	1288	MS,RI	0.44±0.10	-	-	Floral	II
2-Isopropyl-5-methyl-1-heptanol	91337-07-4	Alcohols	30.842	1300.06	-	MS	-	2.20±0.39	-	-	-
Undecanal	112-44-7	Aldehydes	31.204	1307.04	1319	MS,RI	0.61±0.15	-	0.40±0.07	Oil, spicy, sweet	II
11-Methyldodecanol	85763-57-1	Alcohols	31.234	1307.62	-	MS	-	1.92±0.14	-	-	-
2-Hexyl-1-decanol	2425-77-6	Alcohols	31.237	1307.68	-	MS	-	0.96±0.48	-	-	-
4-Methyltetradecane	25117-24-2	Alkanes	31.516	1313.06	-	MS	-	0.63±0.08	-	-	-
2,4-Diethylheptan-1-ol	80192-55-8	Alcohols	31.608	1314.83	-	MS	0.26±0.01	1.47±0.14	-	-	-
Methyl geranoate	1189-09-9	Esters	31.782	1318.19	1322	MS,RI	8.84±1.04	-	3.46±0.31	Floral, fruity	II
4,6,8-Trimethyl-1-nonene	54410-98-9	Ketones	31.797	1318.48	-	MS	-	4.98±0.24	-	-	-
5-(2-methylpropyl)nonane	62185-53-9	Alkanes	32.222	1326.68	-	MS	-	0.96±0.03	-	-	-
7-Methylheptadecan	20959-33-5	Alkanes	32.371	1329.55	-	MS	-	0.39±0.11	-	-	-
Nerolic acid	4613-38-1	Others	33.299	1347.45	1347	MS,RI	-	-	0.66±0.21	-	-
(*E*)-Geranic acid	4698-08-2	Others	33.538	1352.06	1354	MS,RI	9.67±1.26	-	-	Floral	II
2-Methyltetradecane	1560-95-8	Alkanes	34.281	1366.4	-	MS	0.74±0.18	2.23±0.02	0.65±0.09	-	-
Cis-3-Hexenyl hexanoate	31501-11-8	Esters	34.771	1375.85	1376	MS,RI	1.58±0.21	0.70±0.06	1.36±0.05	Green	I
3-Methylidenetridecane	19780-34-8	Alkanes	35.08	1381.81	-	MS	-	1.20±0.05	-	-	-
Hexyl hexanoate	6378-65-0	Esters	35.087	1381.94	1387	MS,RI	0.52±0.07	-	0.99±0.10	Apple-like, peach-like	-
Trans-2-Hexenyl caproate	53398-86-0	Esters	35.264	1385.36	1391	MS,RI	-	-	0.69±0.04	Green, herbal, waxy	I
1-Tridecene	2437-56-1	Alkenes	35.56	1391.07	-	MS	-	0.96±0.11	-	-	-
Tetradecane	629-59-4	Alkanes	36.016	1399.86	1400	MS,RI	3.25±0.59	7.47±0.12	1.48±0.16	Alkane-like	-
α-Ionone	127-41-3	Ketones	37.379	1430.82	1420	MS,RI	0.81±0.21	0.92±0.05	-	Floral, fruity, violet-like	II
Trans-Caryophyllene	87-44-5	Alkenes	37.468	1432.84	1427	MS,RI	-	-	1.07±0.11	Woody	-
Geranylacetone	689-67-8	Ketones	38.508	1456.48	1456	MS,RI	0.59±0.21	1.39±0.13	0.71±0.02	Green, fruity	I
Nonylcyclopentane	2882-98-6	Alkanes	38.639	1459.45	-	MS	0.27±0.10	0.46±0.07	0.19±0.05	-	-
3-Methyltetradecane	18435-22-8	Alkanes	39.425	1477.32	-	MS	0.31±0.08	-	-	-	-
β-Ionone	79-77-6	Ketones	39.757	1484.86	1484	MS,RI	1.85±0.49	4.48±0.16	1.51±0.12	Floral, violet-like	II
Beta-Ionone epoxide	23267-57-4	Ketones	39.893	1487.95	1481	MS,RI	0.16±0.01	1.10±0.17	0.30±0.02	Fruity, sweet, woody	II
Heptadecane	629-78-7	Alkanes	39.991	1490.18	-	MS	0.63±0.13	2.66±0.39	-	Alkane-like	-
Jasmine lactone	25524-95-2	Esters	40.13	1500.93	1489	MS,RI	0.30±0.05	-	2.46±0.11	Floral	II

4-Methylhexadecane	25117-26-4	Alkanes	40.133	1493.41	-	MS	-	0.59±0.10	-	-	-
4,6-Dimethyldodecane	61141-72-8	Alkanes	40.34	1498.11	-	MS	-	0.73±0.15	-	-	-
Pentadecane	629-62-9	Alkanes	40.409	1499.68	1500	MS,RI	1.80±0.42	0.27±0.03	-	Alkane-like	-
2,6-Di-tert-butyl-4-methylphenol	128-37-0	Others	40.519	1503.78	1499	MS,RI	2.78±1.09	1.35±0.08	-	Roast grain	-
α-Farnesene	502-61-4	Alkenes	40.558	1505.32	1507	MS,RI	-	-	8.45±0.71	Woody, sweet	II
Tributyl phosphate	126-73-8	Esters	40.679	1510.09	-	MS	3.44±0.53	1.57±0.10	2.17±0.24	-	-
Delta-cadinene	483-76-1	Alkenes	41.056	1524.95	1516	MS,RI	-	-	0.54±0.03	Herbal, woody	I
8-Hexylpentadecane	13475-75-7	Alkanes	41.183	1529.96	-	MS	-	0.92±0.37	-	-	-
7-Methylhexadecane	26730-20-1	Alkanes	41.247	1532.48	-	MS	0.23±0.07	-	-	-	-
13-Methylheptacosane	15689-72-2	Alkanes	41.619	1547.14	-	MS	0.41±0.06	-	-	-	-
Icosane	112-95-8	Alkanes	41.634	1547.73	-	MS	1.36±0.28	2.35±1.62	-	Alkane-like	-
α-Calacorene	21391-99-1	Alkenes	41.715	1550.93	1541	MS,RI	-	-	0.08±0.01	Woody	-
5-Methylpentadecane	25117-33-3	Alkanes	41.821	1555.1	-	MS	0.38±0.15	-	0.34±0.08	-	-
2,6,10-Trimethyltridecane	3891-99-4	Alkanes	41.86	1556.64	-	MS	0.50±0.10	0.97±0.40	0.70±0.07	-	-
4-Methyldodecane	6117-97-1	Alkanes	41.864	1556.8	-	MS	-	2.94±1.45	-	-	-
4-Methylpentadecane	2801-87-8	Alkanes	41.869	1557	1556	MS,RI	0.78±0.10	-	-	-	-
Nonylcyclohexane	2883-02-5	Alkanes	41.95	1560.19	1566	MS,RI	-	-	0.20±0.08	-	-
Trans-Nerolidol	40716-66-3	Alcohols	42.086	1565.55	1566	MS,RI	2.04±0.35	3.81±0.52	4.71±0.25	Floral	II
Heneicosane	629-94-7	Alkanes	42.14	1567.68	-	MS	0.21±0.06	2.45±1.94	0.34±0.07	-	-
3-Methylpentadecane	2882-96-4	Alkanes	42.294	1573.75	1570	MS,RI	1.28±0.29	1.65±0.44	0.42±0.03	-	-
3-Ethyl-3-methylheptane	17302-01-1	Alkanes	42.393	1577.65	-	MS	-	0.97±0.22	-	-	-
Cis-3-hexenyl benzoate	25152-85-6	Esters	42.399	1577.89	1567	MS,RI	-	-	1.75±0.23	Green	I
Phenyl 3-adamantanecarboxylate	35856-79-2	Esters	42.473	1580.8	-	MS	0.70±0.18	-	-	-	-
Hexyl benzoate	6789-88-4	Esters	42.568	1584.55	1576	MS,RI	-	-	0.63±0.12	Green, woody	I
3-Methylideneundecane	71138-64-2	Alkanes	42.627	1586.87	-	MS	-	0.45±0.14	-	-	-
E-2-Hexenyl benzoate	76841-70-8	Esters	42.749	1591.68	1590	MS,RI	-	-	0.31±0.02	Grern, fruity	I
Ethyl docosanoate	5908-87-2	Esters	42.789	1593.26	-	MS	-	-	0.20±0.02	-	-
1-Pentadecene	13360-61-7	Alkenes	42.802	1593.77	-	MS	-	0.50±0.10	-	-	-
α-Patchoulene	123278-27-3	Alkenes	42.874	1596.61	-	MS	-	-	0.18±0.05	-	-
Nonadecane	629-92-5	Alkanes	42.951	1599.65	-	MS	1.23±0.89	2.17±0.36	-	Alkane-like	-
(*E*)-*γ*-bisabolene	53585-13-0	Alkenes	43.363	1621.54	-	MS	-	-	0.28±0.03	-	-
1,4-Methanobenzocyclodecene, 1,2,3,4,4*α*,5,8,9,12,12*α*-decahydro-	74708-73-9	Alkenes	43.694	1639.23	-	MS	0.37±0.10	-	-	-	-
Methyl jasmonate	1211-29-6	Esters	43.867	1648.48	1652	MS,RI	1.35±0.31	-	1.21±0.10	Jasmine-like	II
Decylcyclopentane	1795-21-7	Alkanes	44.107	1661.3	-	MS	-	0.41±0.09	0.18±0.04	-	-
α-Cadinol	481-34-5	Alcohols	44.231	1667.93	1650	MS,RI	-	-	0.45±0.16	Tar, camphor, greasy	-
2-Methyltetracosane	1560-78-7	Alkanes	44.323	1672.85	-	MS	0.13±0.02	0.43±0.20	-	-	-
2,2',5,5'-Tetramethylbiphenyl	3075-84-1	Others	44.527	1683.75	1663	MS,RI	1.19±0.49	-	-	-	-
Hexadecane	544-76-3	Alkanes	44.615	1688.46	-	MS	0.95±0.69	1.32±0.03	0.62±0.04	Alkane-like	-
1-Iodohexadecane	544-77-4	Alkanes	44.661	1690.91	-	MS	-	0.54±0.00	-	-	-
3,5,24-Trimethyltetracontane	55162-61-3	Alkanes	44.809	1698.82	-	MS	0.85±0.13	2.20±0.06	-	-	-
Pristane	1921-70-6	Alkanes	44.866	1702.28	1703	MS,RI	0.31±0.07	-	-	-	-
3,3',5,5'-Tetramethylbiphenyl	25570-02-9	Others	45.047	1714.06	-	MS	0.97±0.59	-	-	-	-

Dotriacontane	544-85-4	Alkanes	45.518	1744.73	-	MS	-	0.47±0.27	-	-	-
Neryl hexanoate	68310-59-8	Esters	45.602	1750.2	1732	MS,RI	0.25±0.03	-	-	-	-
Ethyl tetradecanoate	124-06-1	Esters	46.236	1791.47	1794	MS,RI	0.16±0.04	-	0.21±0.04	Cream, ether, nuts, oil, pleasant	-
n-Octadecane	593-45-3	Alkanes	46.361	1799.61	1800	MS,RI	0.71±0.40	-	-	Alkane-like	-
Pentacosane	629-99-2	Alkanes	46.367	1800	-	MS	0.12±0.03	-	-	Alkane-like	-
Neophytadiene	504-96-1	Alkenes	46.911	1834.92	1838	MS,RI	0.54±0.13	1.73±0.54	0.58±0.04	-	-
Phytone	502-69-2	Ketones	47.004	1840.89	1835	MS,RI	0.18±0.03	0.53±0.04	0.18±0.02	Fatty	-
Caffeine	58-08-2	Others	47.268	1857.83	1835	MS,RI	7.76±0.72	12.01±3.31	3.90±0.49	-	-
Diisobutyl phthalate	84-69-5	Esters	47.34	1862.45	1870	MS,RI	-	-	0.65±0.07	-	-
3,7,11,15-Tetramethyl-2-hexadecen-1-ol	102608-53-7	Alcohols	47.59	1878.5	1860	MS,RI	0.24±0.05	0.89±0.21	0.26±0.05	-	-
Methyl palmitate	112-39-0	Esters	48.326	1922.71	1926	MS,RI	0.58±0.14	0.81±0.34	1.12±0.14	Oily, waxy, fatty	-
Dibutyl phthalate	84-74-2	Esters	48.947	1957.87	1965	MS,RI	0.45±0.01	1.27±0.71	0.32±0.02	Fruity	-
Ethyl palmitate	628-97-7	Esters	49.52	1990.32	1993	MS,RI	2.41±1.17	1.93±0.52	2.43±0.32	Fatty, sweet, fruity, sour	II
Methyl linoleate	112-63-0	Esters	51.61	2095.47	2087	MS,RI	-	-	0.26±0.04	Oily, fatty, woody	-
8,11,14-Docosatrienoic acid, methyl ester	56847-02-0	Esters	51.736	2102.23	-	MS	-	-	0.28±0.04	-	-
Phytol	150-86-7	Alcohols	51.904	2112.94	2128	MS,RI	1.00±0.18	3.41±0.96	0.95±0.08	Floral	II
Ethyl linoleate	544-35-4	Esters	52.792	2169.53	2164	MS,RI	0.79±0.16	0.47±0.05	0.70±0.10	Waxy, oily, fatty	-
Ethyl linolenate	1191-41-9	Esters	52.901	2176.48	2171	MS,RI	0.58±0.15	0.43±0.11	0.59±0.15	Fatty, waxy	-

Note: The reference retention indices are sourced from the NIST Chemistry WebBook website and relevant literature. The aroma descriptions are derived from the VCF, Flavornet websites and related literature.
